# Unfair older patients restriction in cancer drug trials in mainland China and corresponding solution

**DOI:** 10.1186/s12877-023-03886-2

**Published:** 2023-03-30

**Authors:** Huiyao Huang, Yu Tang, Dawei Wu, Xinyu Meng, Shuhang Wang, Jun Wang, Yue Yu, Yuan Fang, Hong Fang, Qi Zhu, Ning Li, Binghe Xu, Yan Sun, Jie He

**Affiliations:** 1 Clinical Trial Office, National Cancer Center/National Clinical Research Center for Cancer/Cancer Hospital, Chinese Academy of Medical Sciences, Peking Union Medical College, 17 South Panjiayuan Lane, Chaoyang District, Beijing, 100021 People’s Republic of China; 2 School of Population and Global Health, the University of Melbourne, Victoria, 3010 Australia; 3 Center for Drug Evaluation, National Medical Products Administration, Beijing, 100022 China; 4 School of Basic Medicine and Clinical Pharmacy, China Pharmaceutical University, Nanjing, 211198 China; 5 Department of Thoracic Surgery, National Cancer Center/National Clinical Research Center for Cancer/Cancer Hospital, Chinese Academy of Medical Sciences and Peking Union Medical College, Beijing, 100021 China

**Keywords:** Neoplasms, Clinical trial, Age disparity, China

## Abstract

**Background:**

Older adults are a growing segment of oncology population in China and beyong. However, older cancer patients were vastly underrepresented in clinical trial. To facilitate that all patients with cancer have equal access to the cutting edging treatment and receive evidence-based medication in mainland China, it’s of particular importance to fully grasp the proportion of upper age restriction in cancer clinical trials, as well as associated factors.

**Methods:**

Based on clinical trials registered on the China Food and Drug Administration Registration and Information Disclosure Platform, we sought to characterize the overall proportion and trajectory of upper age-restriction among registered cancer drug trials in mainland China from 2009 to 2021, and potential influencing factors were determined by multivariate logistic regression.

**Results:**

According to the 3485 trials, upper age restriction proportion of cancer drug trials for patients over 65 years and 75 years was 18.8% (95% CI = 17.5%-20.1%) and 56.5% (95% CI = 51.3%-54.6%), respectively. Phase IV trials, international multicenter trials, or trials initiated by global companies seldom excluded patients over 65 years compared with phase I trials, domestic trials and trials initiated by Chinese enterprise, similar for 75 years and above. Both of 65 and 75 years old age limit sponsored by domestic enterprises showed slowly downward trend, while no such trend was observed for that of foreign companies. Solution to upper age eligibility of cancer drug trials was also provided.

**Conclusions:**

Although there is a certain downward trend, use of eligibility criteria that explicitly exclude older cancer patients in mainland China was remarkably high, especially for trials initiated by domestic enterprise, domestic trials and early-phase trials. Action is urgently needed to promote treatment equity in the older patients while obtaining adequate evidence in clinical trials.

## Introduction

As the population ages worldwide, the number of new cancer cases is expected to double and estimated to reach 14 million among adults aged 65 years and above by 2035, representing 58% of the global cancer incidence [1]. Undoubtedly, older patients are the top priority in cancer treatment and control. Clinical trial and cancer care are closely intertwined, it is widely recognized that clinical trial is an alternative treatment, especially benefits cancer patients without standard treatment [2], thus, it is unfair to exclude older cancer patients from clinical trials solely on the basis of age. Remarkably, older patients can respond differently from younger patients to drug therapy, and these differences can be most pronounced in those over age 75 [3]. To ensure safe and effective medication for older patients, the data of the older patients in the clinical trial process should be highly valued. However, older patients are extremely underrepresented in cancer clinical trials despite representing a growing segment of cancer patients [4, 5], which is closely related to upper age limits, it determines the participation qualifications of geriatric [6].

So as early as 1993, ICH has specially issued guideline on studies in support of geriatric populations and pinpointed the representativeness of aged population as a general principle [7]. Unfortunately, substantial progress of regulatory guidances, potential specific strategies, even essential proportion of age restriction in cancer clinical trials remains dismall worldwide, except in the United States [6, 8, 9] Therefore, foucus on mainland China, the country with the largest global older cancer population, we pioneered to explore current proportion, time trend and asscociated factors of upper age limits in registered cancer drug trials, and we sought to analyze the reasons behind the important scientific and social issues of upper age restriction in clinical trials, and finanly put forward reasonable suggestions on age restrictions of older patients in different stages of clinical development of new drugs.

## Methods

Data query was performed based on the national authoritative database, the China Food and Drug Administration (CFDA) Registration and Information Disclosure Platform for Clinical Trials, which includes all the drug clinical trials for registration purpose in mainland China [10]. A total of 9752 drug trials were registered in mainland China, and 3823 cancer trials were identified through indication by keywords and manual review. Detailed data process was described in previous study [11]. In this study, the trial was further excluded if meet any of the following criteria: a) study phase wasn’t clear, b) study scope unspecified, c) effect of tested drug was cancer prevention, d) tested drug was generics, e) age eligibility was missing, f) trial mainly targeted on pediatric cancer patients. Both trial screening and identification were performed independently by two individuals. In case of disagreement, a third-party oncologist was invited to arbitrate until the decision was unified.

The primary endpoint of the study was 65-year age restriction proportion, and the secondary endpoint was 75-year age restriction proportion. The trial was considered to have an age restriction of 65 years if an upper age cutoff of 65 years or younger was provided, and related definition for age restriction of 75 years was similar. Statistics on 65-year and 75-year age restriction proportion were conducted respectively. Since China joined ICH in 2017, domestic enterprises and foreign enterprises may have different awareness of the age restriction issue, thus time trend analysis of age restriction incidence by sponsor type was analyzed.

Chi-square test was used to test the association between age restriction proportion and the following variables: study phase, trial scope, sponsor type, cancer type, drug effect, drug attribute, drug molecule. All of the above variables were included as explanatory variables in the multivariate logistic regression. The model was fitted by stepwise regression, with the input and output thresholds of variables as 0.05 and 0.10 respectively. C-statistics was calculated to assess the performance of the logistic regression, with 0.7 being the threshold of good prediction ability.

In addition, further calculations of age restriction proportion for different cancer types with more than 20 drug trials was performed. Meanwhile, incidence proportion of older patients over 64 years and over 74 years for each cancer type were acquired from 2018 China Cancer Registry Annual Report [12]. 10-year time trend on age restriction by sponsor type proportion was predicted using simple regression model. To check for the robustness, sensitivity analyses of 10-year time trend on age restriction after adjusting by study phase, study scope and cancer type were further performed. Statistical analysis were conducted using two-tailed test at a significance level of 0.05 and with SAS statistical software, version 9.4 (SAS Institute, Cary/NC, US). Institutional review board approval was not required as all data were publicly available without any use of protected health information.

## Results

Three thousand four hundred eighty-five cancer trials were included in the final analysis, 85.2% of which were targeted on solid tumor, accounting for 91.2% of all registered cancer drug trials in mainland China. In 3485 cancer trials, 2886 (82.8%) trials were domestic while 599 (17.2%) were international, and 2792 (80.2%) trials were sponsored by domestic industries while 691 (19.8%) trials were sponsored by global enterprises. In terms of study phase, phase I trials accounted for the largest proportion (44.7%), followed by phase III trials (22.6%) and phase II trials (19.1%). The majority trials were tested on innovative drugs (81.6%) and therapeutic medication (93.9%). Regarding drug molecule, chemistry medicine was most frequently tested (60.8%), followed by biological products (38.2%). For detailed characteristics of included trials was displayed in Table 1.

**Table 1:** Basic characteristics of included 3485 cancer drug trials in China

Variables	No	%
**Study phase**		
Phase I	1557	44.7%
Phase II	666	19.1%
Phase III	787	22.6%
Phase IV	41	1.2%
**Study scope**		
Domestic trials	2886	82.8%
International multicenter trial	599	17.2%
**Sponsor type**^**a**^		
Domestic industry	2792	80.2%
Global industry	691	19.8%
**Cancer type**		
Solid tumor	2969	85.2%
Blood tumor	516	14.8%
**Drug effect**		
Therapeutic medication	3271	93.9%
Diagnosis medication	2	100.0%
Adjuvant medication	212	6.1%
**Drug type**		
Innovative drug	2843	81.6%
Biosimilar	134	3.8%
Generics	508	14.6%
**Drug molecule**		
Chemistry medicine	2119	60.8%
Biological Products	1333	38.2%
TCM/NM^b^	33	0.9%

^a^Missing cases were not included

^b^Traditional Chinese Medicine/Natural Medicine

There were 18.8% (95% confidence interval [CI]: 17.5%-20.1%) and 56.5% (95% CI: 51.3%-54.6%) of cancer drug trials in mainland China have an upper age restriction on 65 years and 75 years, respectively. Except for variable cancer type, other explored variables were shown to have associations with proportion of age restriction on both 65 years and 75 years in the univariate analysis (*P* < 0.001). There were huge disparities in 65 years old restriction between phase IV trials (2.4%, 95% CI: 0.1%-14.4%) and phase I trials (24.7%, 95% CI: 22.6%-26.9%), international multicenter trials (0.5%, 95% CI: 0.2%-1.5%) and domestic trials (22.6%, 95% CI: 21.1%-24.2%) as well as trials initiated by global companies (3.8%, 95% CI: 2.6%-5.5%) and Chinese enterprise (22.5%, 95% CI: 21.0%-24.1%). Similar age restriction disparities among above groups were also observed for patients over age 75 years. As for anticancer medications, innovative drugs, and biological products had a relatively low proportion of both upper age limit on 65 years and 75 years (Table 2).

**Table 2:** Univariate analysis of factors associated with age restriction proportion of cancer drug trials in China

**Variables**	**Age restriction of 65 years**			**Age restriction of 75 years**		
	No	Yes	*P* value	No	Yes	*P* value
**Study phase**			< 0.001			< 0.001
Phase I	75.3%	24.7%		33.8%	66.2%	
Phase II	97.4%	2.6%		58.9%	41.1%	
Phase III	97.8%	2.2%		68.7%	31.3%	
Phase IV	97.6%	2.4%		68.3%	31.7%	
**Study scope**			< 0.001			< 0.001
Domestic trials	77.4%	22.6%		37.2%	62.8%	
International multicenter trial	99.5%	0.5%		94.2%	5.8%	
**Sponsor type**			< 0.001			< 0.001
Chinese company	77.5%	22.5%		36.1%	63.9%	
Global company	96.2%	3.8%		91.2%	8.8%	
**Cancer type**			0.392			0.155
Solid tumor	81.4%	18.6%		46.2%	53.8%	
Blood tumor	79.8%	20.2%		51.9%	48.1%	
**Drug effect**			< 0.001			< 0.001
Therapeutic medication	93.9%	16.8%		48.5%	51.5%	
Diagnosis medication	100.0%	0.0%		50.0%	50.0%	
Adjuvant medication	50.9%	49.1%		24.1%	75.9%	
**Drug type**			< 0.001			< 0.001
Innovative drug	87.9%	12.1%		50.9%	49.1%	
Biosimilar	58.2%	41.8%		16.4%	83.6%	
Generics	49.6%	50.4%		33.7%	66.3%	
**Drug molecule**			< 0.001			< 0.001
Chemistry medicine	74.6%	25.4%		47.1%	52.9%	
Biological Products	91.7%	8.3%		47.9%	52.1%	
TCM/NM	84.8%	15.2%		12.1%	87.9%	

**Fig. 1:**
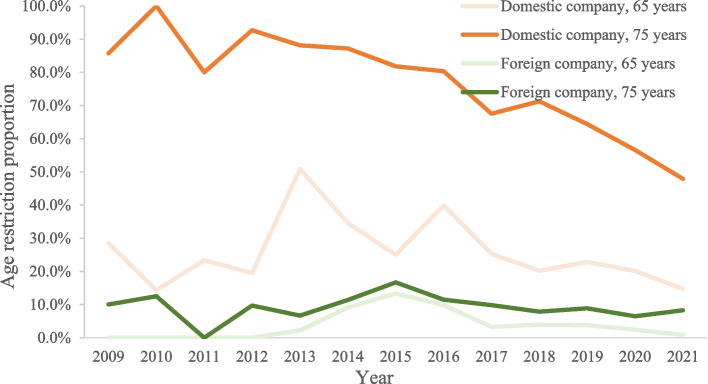
Annual age restriction proportion by sponsor type, 2009–2021

On the whole, the proportion of age restrictions in domestic companies is higher than that of global companies, whether on 65 or 75 years old (Fig. 1). In terms of changing trend, the age limit of trials sponsored by domestic enterprises was slowly downward, from 28.6% in the proportion of the 65-year-old age restreiction in 2009 to 14.8% in 2021 (*F* = 10.24, *P* = 0.0085), from 85.7% in the proportion of the 75-year-old age restriction in 2009 to 47.9% in 2021 (*F* = 93.25, *P* < 0.0001). Sensitivity analysis after adjusting study phase, study scope, cancer type were showed similar results. No trend was observed in the proportion of age restriction in trials iniatiated by foreign companies, including on 65 (*F* = 0.52, *P* = 0.487) and 75 years old (*F* = 1.02, *P* = 0.334).

Overall, significant variables included in the multivariate regression model were broadly consistent with the univariate analysis, except for sponsor type was excluded in the multivariate analysis of 65-year age restriction proportion. The p-value of corresponding Hosmer–Lemeshow goodness-of-fit test for 65-year and 75-year age restriction model was 0.122 and 0.317, and the corresponding c-statistics was 0.862 and 0.772, respectively, suggesting a good prediction ability of our models.

According to Table 3, Phase II (OR = 0.08, 95%CI:0.05–0.14; OR = 0.42, 95%CI:0.34–0.52), phase III trials (OR = 0.07, 95%CI:0.04–0.13; OR = 0.66, 95%CI: 0.52–0.84) and phase IV (OR = 0.06, 95%CI: 0.01–0.46; OR = 0.34, 95%CI: 0.16, 0.75) were less likely to have upper age limit on both 65 years and 75 years old. In contrast, domestic trials (OR = 12.52, 95%CI: 3.89–40.31; OR = 8.69, 95%CI: 5.89–12.86), biosimilars (OR = 15.50, 95%CI: 9.35–25.67; OR = 2.78, 95%CI: 1.68–4.61) or TCM/NM (OR = 10.80, 95%CI: 3.54–32.95; OR = 6.91, 95%CI: 2.16–22.13) were more likely to set up age limits to 65 years and 75 years old. Besides, chemistry medicine (OR = 4.64, 95%CI: 3.73–6.38) and adjuvant medication trials (OR = 5.07, 95%CI: 3.47–7.40; OR = 2.19, 95%CI: 1.53–3.14) was positively correlated with 65-year and 75-year age restriction.

**Table 3:** Multivariable analysis of factors predicting age restriction of cancer trials among older patients

**Variables**	**65 + patients**			**75 + patients**		
	**OR (95%CI)**		***p* value**	**OR (95%CI)**		***p* value**
**Study phase**						
Phase I	1			1		
Phase II	0.08	(0.05–0.14)	< 0.001	0.42	(0.34–0.52)	< 0.001
Phase III	0.07	(0.04–0.13)	< 0.001	0.66	(0.52–0.84)	0.009
Phase IV	0.06	(0.01,0.46)	0.0065	0.34	(0.16–0.75)	0.0076
**Study scope**						
International multicenter trial	1			1		
Domestic trials	12.52	(3.89–40.31)	< 0.001	8.69	(5.89–12.82)	< 0.001
**Sponsor type**						
Global industry				1		
Domestic industry				6.15	(4.51–8.38)	< 0.001
**Drug effect**						
Therapeutic medication	1			1		
Diagnosis medication	< 0.01		0	1.76	(0.08–28.03)	0.72
Adjuvant medication	5.07	(3.47–7.40)	< 0.001	2.19	(1.53–3.14)	< 0.001
**Drug type**						
Innovative drug	1			1		
Biosimilar	15.50	(9.35–25.67)	< 0.001	2.78	(1.68–4.61)	< 0.001
Generics	1.98	(1.1–3.56)	0.023	1.83	(1.07–3.12)	0.03
**Drug molecule**						
Biological Products	1			1		
Chemistry medicine	4.64	(3.37–6.38)	< 0.001	0.84	(0.70–1.00)	0.05
TCM/NM	10.80	(3.54–32.95)	< 0.001	6.91	(2.16–22.13)	0.001

**Fig. 2:**
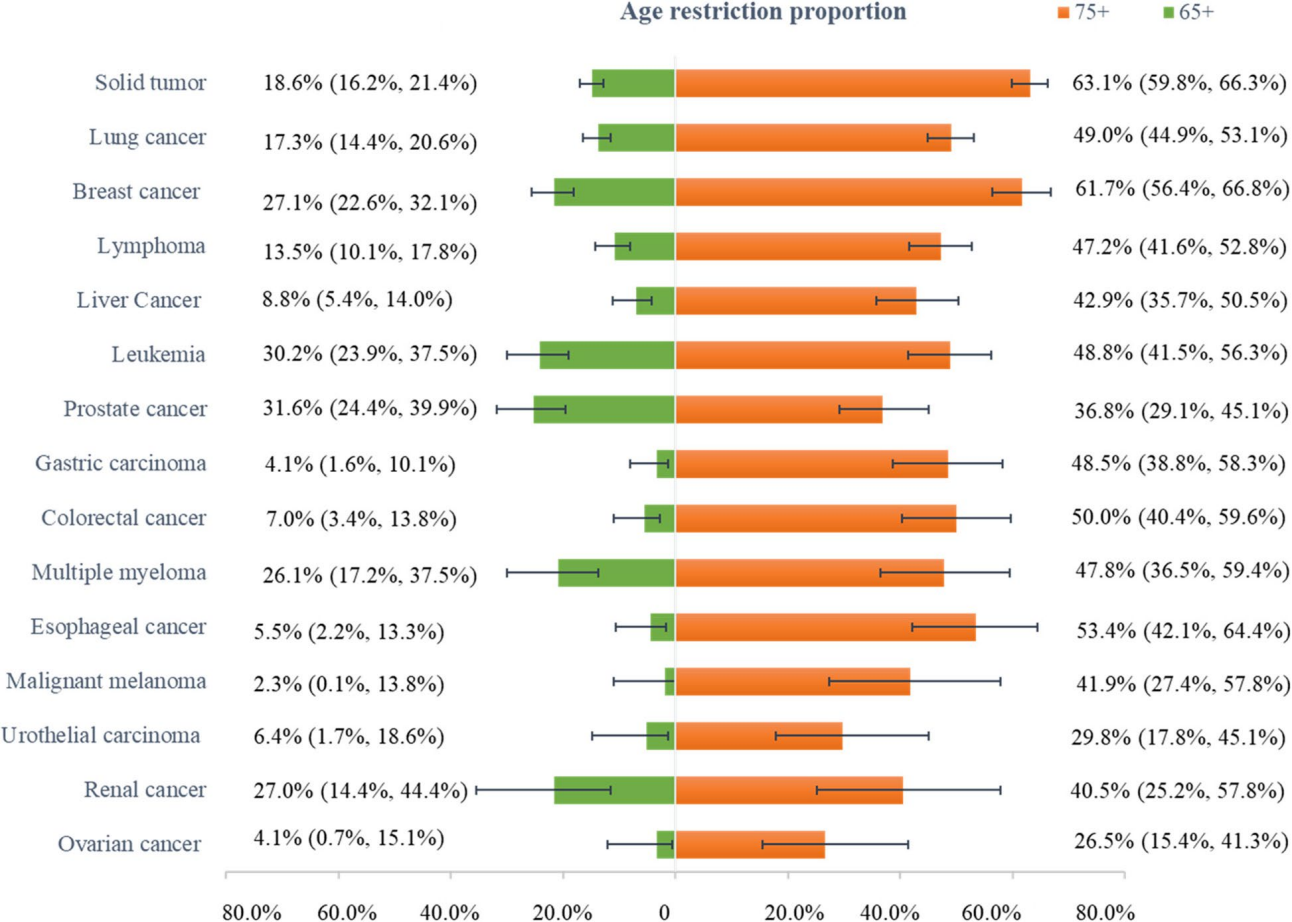
Age restriction proportion of various cancer drug trials in China. Note: The data in the above figure was the proportion and its confidence interval of upper age restriction for older patients over 65 years and 75 years by diffirent cancer type

Age restriction proportion of various cancer type was further explored and displayed in Fig. 2. Prostate cancer, leukemia and renal cancer ranked top three of 65-year age restriction, while malignant melanoma, gastric carcinoma and ovarian cancer seldomly excluded patients over 65 years old. 75-year age restriction was most common in trials targeting on solid tumor, breast cancer and esophageal cancer, with respective proportion amounting to 63.1% (95%CI:59.8%-66.3%), 61.7% (95%CI: 56.4%-66.8%) and 53.4% (95%CI: 42.1%-64.4%), followed by colorectal 50.0% (95%CI: 40.4%-59.6%), lung 49.0% (95%CI: 44.9%-53.1%), leukemia 48.8% (95%CI: 41.5%-56.3%) and gastric carcinoma 48.5% (95%CI: 38.8%-58.3%).

## Discussion

To our knowledge, this represents the first study providing such comprehensive analysis of the proportion of and factors associated with upper age restriction among cancer drug clinical trials in mainland China. We found that upper age restriction proportion in cancer drug trials was remarkably high in China, with respective age restriction proportion for patients over 65 years and 75 years being 18.8% and 56.5%, which appears a great difference compared with reported data from the United States, 75-year age restriction proportion < 5.5% [13]. Moreover, focus on pharmaceutical company only in mainland China, a higher age restriction proportion appears in trials sponsored by local company with 22.5% than global company with 3.5%.

Why are cancer drug trials restrictive for older patients? Practically specking, eligibility criteria of clinical trials are jointly enacted by the sponsor and the physician under regulatory and ethical requirement. Without clear regulatory guidelines, all parties have certain incentives to actively exclude or passively accept the exclusion of older patients from clinical trials, ethics committee from the perpectives of protecting those vulnerable participants, sponsors from the holistic risk benefit trade-offs, and physicians from the actual difficulty and risk of management. Therefore, in view of this kind of scientific and equity issue, regulatory guidance is required to clear out the direction and grasp intensity in order to ensure health care fairness.

Why does the United States have looser age restrictions than China, even foreign enterprises in China have less age restrictions than domestic enterprises? The reason is United States Food and Drug Administration (FDA) is leading the way in handling health care fairness issue. The FDA proposed a decade ago that should not arbitrarily include upper age cutoff and emphasized the importance of including older patients in issued guidance [14, 15]. Meanwhile, the FDA has also required that the package inserts of approved products include “Geriatric Use” subsection that provides pertinent information about the drug's risk–benefit profile in the older patients [16]. These specific guidance and persistent efforts laid the foundation of global clinical trial development, increased age diversity inclusion in international multicenter trials and trials initiated by global companies. China, in contrast, besides of ICH E7 issued in 1993 [7], no snew guidelines were released until Center for Drug Evaluation (CDE) released “Clinical Value-Oriented Guiding Principles for Clinical Development of Anti-tumor Drugs” in 2021 [17].

**Fig. 3:**
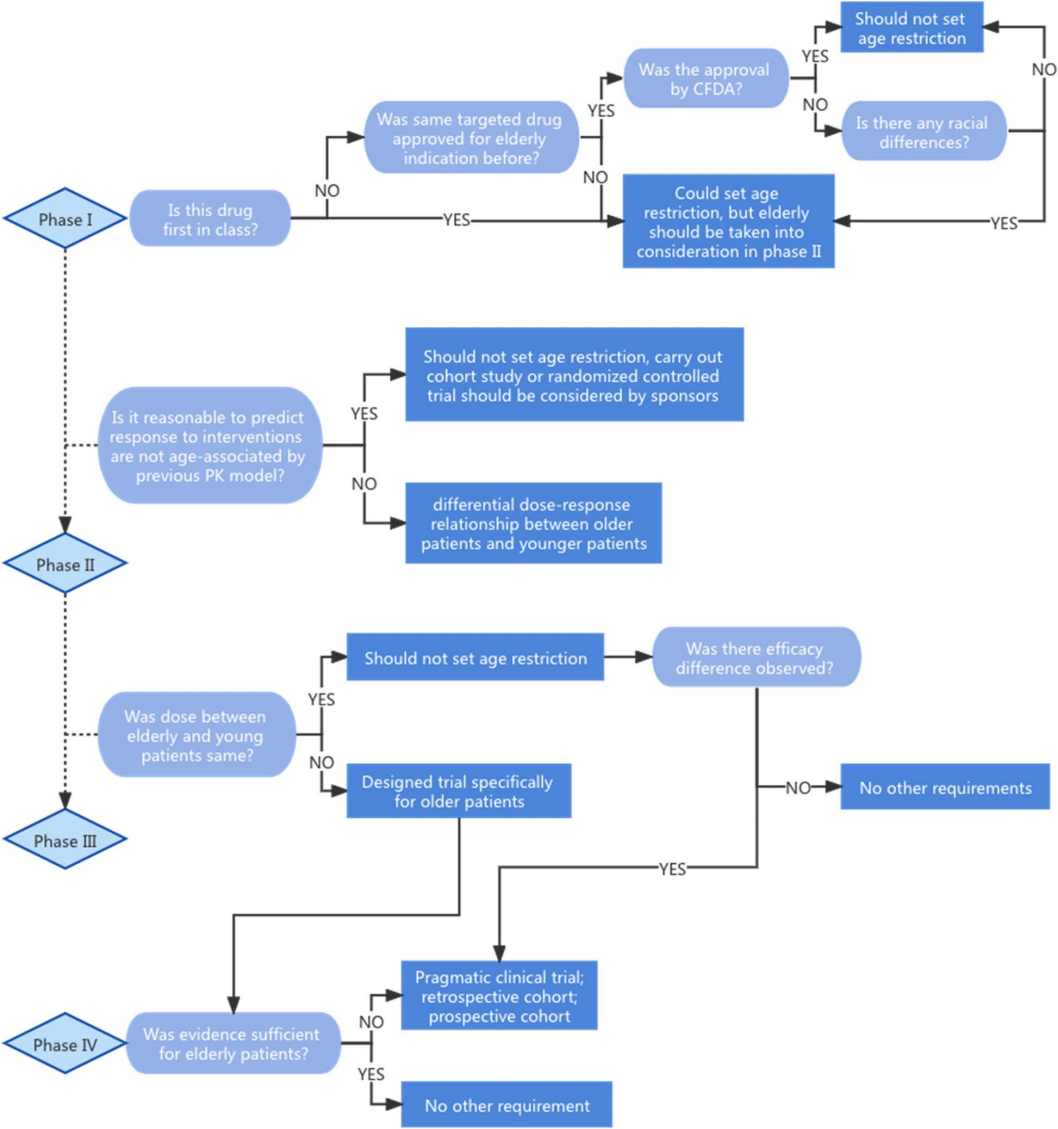
Solution to older eligibility of clinical cancer trials

What is a better solution to upper age eligibility of clinical trials that balance safety, fairness and scientificity? Overall, we believe that the underlying principle is to take into account all available evidence on investigational drug itself and comechanistic drugs, from preclinical, pharmacokinetic, pharmacodynamic to clinical data, and then scientifically determine whether older patients are at higher risk than younger patients. If not, there should not set any age restriction at any stage of cancner clinical trials, and group over 65 years should be representative (Fig. 3). However, if yes, synchronous inclusion of older patients can’t be implemented, we should find new ways to satisfy the needs of older patients to participate in trials and their medication evidence is sufficient. For example, when exploratory study shows difference in dose among older patients, then we suggest that it is best to prescribe a specific confirmatory trial for older patients, or carry out a pragmatic clinical trial, prospective cohort or retrospective study after drug approval. If there is no difference in dose but potentially large difference in effect size or even directipn among older patients, we may recommend to design an adaptive trial in phase III or later in phase IV.

As the proportion of older cancer cases has reached to 44.9% and is predicted to rise with the life expectancy increasing in China [18], we believe it is imperative to increase older patients’ participation in clinical trials. Our study is a call to action, particular emphasis must be placed on older cancer participants in clinical trials in China to ensure trials’ scientificity, fairness and availability, especially for those late phase trials, as well as those cancer types with older adults representing the majority of patients receiving treatment, such as prostate cancers, esophageal cancers and lung cancers. Additional risk in older cancer patients should be comprehensively analysed before setting upper age limits, If it is limited, further studies on the safety and efficacy of medication in the older should be designed. Most importantly, regulatory strategy should be advanced to develop clearer guidance for broadening age eligibility criteria range and expanding older patients inclusion in cancer clinical trials in China.

Based on national authoritative database, this study pioneered to shed a bright light on the overall proportion as well as its trajectory of upper age restriction in cancer drug trials in China, pointing out a imperative call for action, policy-makers and related stakeholders should react to this issue and make corresponding efforts to ensure that all patients with cancer, including those aged population, have equal opportunity to receive high quality, up to date and evidence-based care. As for the limitations of this study, it manifest mainly in two aspects. The first one is our study was confined to drug trials for registration purpose in China. Trials registered outside China and investigator-initiated trials were not involved. The second one is except age restriction, a wide range of barriers could potentially influence clinical trial participation of older cancer patients. Further patient-level studies should be carried out to fully grasp actual participation rate and other barriers to older participation in clinical trials in China.

## Data Availability

All statistics can be obtained from the China Food and Drug Administration Registration and Information Disclosure Platform with administrative permissions. The datasets generated during the current study are not publicly available due to administrative permission required but are available from the corresponding author on reasonable request, thus, raw data prefer not to be shared.
